# Ultrasound vs. Reality: A Multi-centre Study of Real-World Imaging Practices in Suspected Appendicitis in the United Kingdom

**DOI:** 10.7759/cureus.87445

**Published:** 2025-07-07

**Authors:** Naman Vashistha, Soumya Singh, Farooq Ali

**Affiliations:** 1 Internal Medicine, Manchester University NHS Foundation Trust, Manchester, GBR; 2 Geriatrics, Morriston Hospital, Swansea, GBR; 3 Radiology, Manchester University NHS Foundation Trust, Manchester, GBR

**Keywords:** appendectomy, appendicitis, appendicitis diagnostic accuracy, diagnostic approach of appendicitis, imaging in appendicitis cases, imaging of appendix, imaging preference in appendicitis, ultrasound efficacy, ultrasound efficacy in appendicitis

## Abstract

Background: Appendicitis is one of the leading causes for emergency surgery in the United Kingdom (UK), with approximately 50,000 appendectomies performed annually. While imaging plays an increasingly important role in diagnosis, the lack of specific, unified guidelines guiding the utilisation of various imaging modalities causes ambiguity in how and when various modalities should be used.

Objective: This study aimed to evaluate how various imaging modalities are being used in day-to-day practice, the practical aspects, the challenges, and the benefits of one scan over the others. This was done by comparing the diagnostic accuracy of various scans, such as ultrasound (US), CT, and MRI scans in suspected appendicitis cases.

Methods: We retrospectively reviewed 200 recent appendectomy cases across two major hospitals in Manchester. After excluding 70 patients who either had no preoperative imaging or were diagnosed with something other than appendicitis, we analysed the radiology and histopathology reports of the remaining 130 patients. Special attention was given to the subgroup of 52 patients who initially underwent US.

Results: US had a sensitivity (Sn) of 56.25%, with 21 missed diagnoses later confirmed via CT, MRI, or histopathology. US should be commonly used for those under 18, but surprisingly, approximately two-thirds of US cases were adults, representing an inclination to use US as an initial investigation. In the under-18 group, US Sn was 80%, dropping to just 37.5% in the 18-29-year age group. US was not able to visualise the appendix in 34% (n=18) of patients, missing out on many appendicitis diagnoses. The CT scan was the most utilised scan and had a sensitivity of nearly 99%. The MRI scan, although limited in numbers, had a sensitivity of 100%.

Conclusion: While US offers benefits such as speed and no radiation exposure, its diagnostic reliability varies with the patient’s body habitus and the operator’s skills. The appendix was not identified in a good proportion of patients; however, when the appendix was clearly visualised, it was associated with a better sensitivity. The CT scan was the most utilised scan. It also proved to have excellent sensitivity and is quicker to perform compared to an MRI scan. The MRI scan provides CT equivalent sensitivity but without exposure to harmful radiation. However, it was being underutilised due to limitations in practicality and availability. We routinely request these scans to support the diagnosis of appendicitis, but it is important to understand their diagnostic value, limitations, and when they are most appropriately used.

## Introduction

Appendicitis continues to be a major cause of emergency surgical admissions, and timely diagnosis remains critical to reducing complications. In the United Kingdom (UK) alone, over 50,000 appendectomies are performed each year [1]. Although clinical evaluation still forms the foundation of assessment, imaging is often required to support or clarify diagnosis, especially in equivocal presentations.

Despite the growing reliance on imaging, current guidelines do not explicitly outline which imaging modality should be used first or when to escalate; this may be because such decisions are often case-dependent. However, clearer recommendations could still be helpful in standardising practice. Ultrasound (US) is often favoured as a first-line tool due to its non-invasive nature, wide availability, and absence of radiation; however, it has variable reported reliability. Additionally, the use of advanced imaging techniques, such as CT and MRI, is now common in appendicitis cases. Thus, it is important to understand how these are being used in real-world settings and to consider the practical factors involved in their application. This study examines real-world imaging use in appendicitis cases, with a focus on the role of US. By comparing diagnostic outcomes across US, computed tomography (CT), and magnetic resonance imaging (MRI), including sensitivity (Sn), negative appendectomy rates (NAR), among other metrics, we aim to better understand their relative contributions and identify areas where diagnostic pathways can be improved.

## Materials and methods

Study design

This is a retrospective review looking at 200 consecutive appendectomy cases from across hospitals under the Manchester University NHS Foundation Trust. The data come from two of the biggest hospitals in Manchester, namely, Manchester Royal Infirmary and Wythenshawe Hospital, and include both adult and paediatric cases. These cases presented to the hospital from September 2022 to January 2023. The aim was to look at how imaging was used in real-life settings and how well it matched up with histopathology reports found after surgery. Descriptive statistics, including means, were calculated using Microsoft Excel (Microsoft® Corp., Redmond, WA). Sensitivity (Sn), specificity (Sp), positive predictive value (PPV), and negative predictive value (NPV) were calculated using standard formulas. Negative appendectomy rates (NAR) were calculated as the proportion of non-appendicitis cases among all appendectomies performed based on positive imaging findings. Ninety-five percent confidence intervals (CIs) for sensitivity were estimated using the Clopper-Pearson method.

Inclusion and exclusion criteria

**Figure 1:**
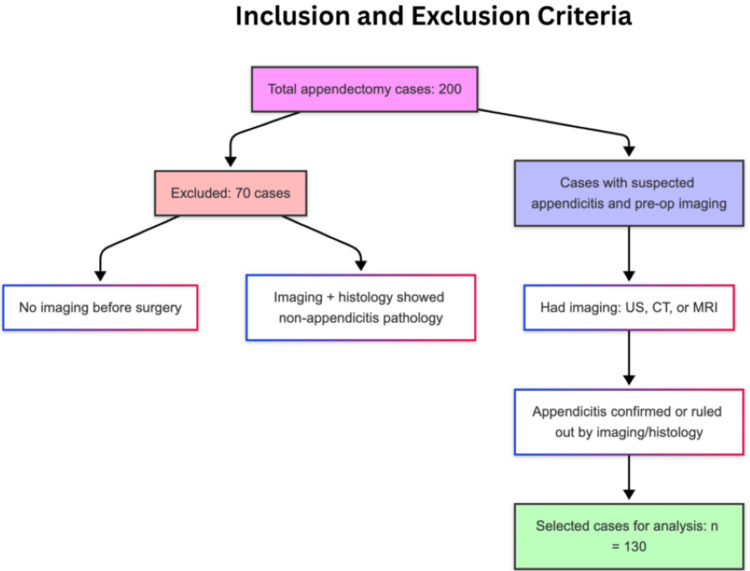
Flowchart presentation of the inclusion and exclusion criteria. CT: computed tomography; MRI: magnetic resonance imaging; US: ultrasound; NAR: negative appendectomy rate Image Credit: The figure is original and derived from the data collected in the present study.

Out of the 200 appendectomy cases, we focused on those who were being worked up for suspected appendicitis and had some form of imaging before surgery (n=130). The remaining 70 patients (35%) either did not have any imaging before surgery or turned out to be something other than appendicitis, so they were excluded from the analysis. Most of the cases without imaging were likely straightforward clinical diagnoses, where the decision to operate was based on strong clinical suspicion without the need for a scan. To keep the study focused, we applied the following criteria (Figure 1).

Data collection

Demographic data, such as age and sex, were collected for all cases, along with details on whether imaging was performed prior to surgery. For those who had imaging, the type of scan(s) used was recorded. In cases where more than one imaging modality was used, the specific scans and the order in which they were performed were noted. The reports from these scans, namely, US, CT, and MRI, were then compared with the post-surgical histopathology results for accuracy and correlation.

Subgroup analysis

We paid closer attention to a group of 52 patients who had a US as their first imaging test. We looked at how many of these patients had their diagnosis confirmed on US, how many were missed and picked up later, and whether more imaging was needed. We also broke this down by age and gender to see if any patterns stood out. A comparison of US sensitivities before and after excluding non-visualised cases was carried out with Fisher's exact test.

Outcome measures

The main thing we looked at was how sensitive each type of scan was when it came to picking up appendicitis, using the histopathology result after surgery as the gold standard. We also noted how many patients had surgery based on a positive radiology report but turned out not to have appendicitis (NAR). Specificity, PPV, and NPV, if calculated, were only included at places where sufficiently powered by adequate numbers.

## Results

CT scan findings

The study included 130 cases, with 91 CT scans, six MRI scans, and 52 US scans analysed. CT scans were the most commonly used imaging tool in our cohort. It showed excellent sensitivity at 0.98 (95% CI: 0.93-0.99) and a strong PPV of 0.97, but specificity and NPV were underpowered and not reliable due to the very low number of cases in the respective calculations. Out of all patients who underwent an appendectomy after a positive CT scan finding (n=89), two of them did not have proven appendicitis on post-operative histopathology report (false positive), giving a NAR of just 2.24% (Table 1).

**Table 1:** Summary of CT scan results.

CT Findings	Number of Case/Value
Total CT scans	91
True Positives (TP)	87
False Negatives (FN)	1
True Negatives (TN)	1
False Positives (FP)	2
Sensitivity (%)	98.86
Negative Appendectomy Rate (%)	2.24

**Figure 2:**
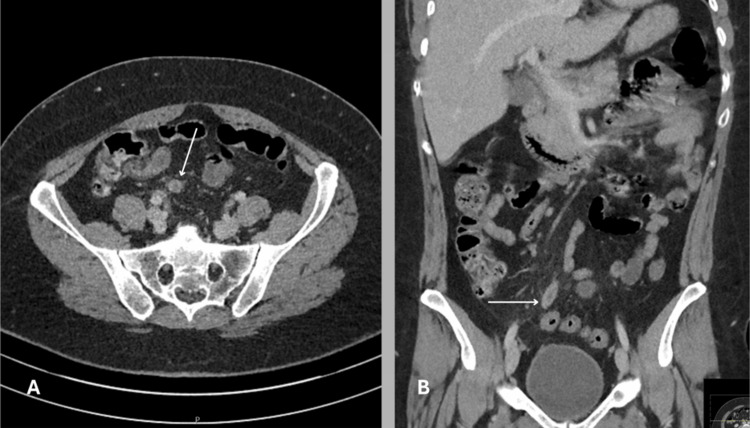
CT scan of a 25-year-old female from the study showing classic signs of acute appendicitis. Typical CT findings in appendicitis include a distended, thick-walled appendix with surrounding fat stranding indicating peri-appendiceal inflammation. A) Axial cross-sectional view showing the inflamed appendix with fat stranding (arrow). B) Coronal view confirming the same finding (arrow). Image Credit: The figure is original and derived from the data collected in the present study.

CT was the most frequently used scan type, which clearly performed well, with high sensitivity and a low NAR, supporting its role as one of the most reliable imaging methods in this setting. A CT scan from one of the patients in the study highlights the high resolution and excellent contrast that CT imaging provides. As a cross-sectional modality, it captures multiple planes in fine detail, typically with slice thickness as low as 0.5 mm, allowing for a comprehensive evaluation (Figure 2).

MRI findings

MRI was used in only six patients out of the 130, so from the outset, we know the sample is too small to draw strong conclusions. Still, within this small group, MRI performed really well. Five of the total six cases were correctly picked up as appendicitis (true positives), and none were missed (false negatives), giving it a sensitivity of 100%. That said, one of these six patients did not have a proven appendicitis on histopathology (false positive) despite MRI suggesting appendicitis, which gives a NAR of 16.66%. The reason that so few MRIs were done probably comes down to practical limitations. MRI is not always easily available in acute settings, takes longer, and is often reserved for specific cases such as pregnancy or when CT is not ideal. Thus, while the accuracy looks great on paper, the numbers are just too small to rely on, and this group is clearly underpowered for proper analysis. A table summarising the results from the MRI scans can be seen in Table 2.

**Table 2:** Summary of MRI scan results.

MRI Findings	Number of Cases/Value
Total MRI Scans	6
True Positives (TP)	5
False Negatives (FN)	0
True Negative (TN)	0
False Positive (FP)	1
Sensitivity (%)	100
Negative Appendectomy Rate (%)	16.66

**Figure 3:**
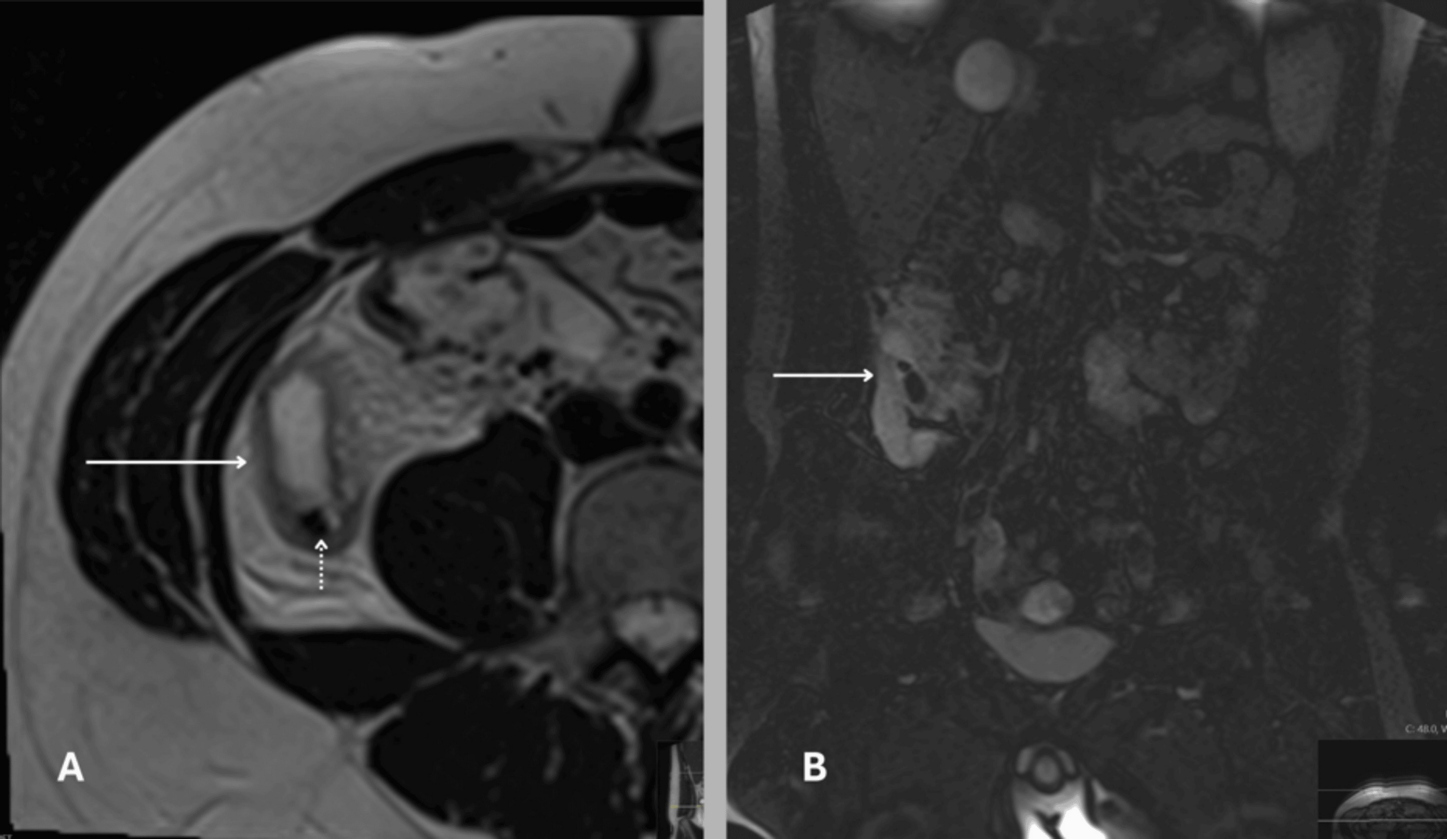
MRI scan of a 23-year-old man from the study showing typical signs of acute appendicitis. A) Axial T2-weighted image showing a distended, thick-walled appendix (arrow), with a surrounding hyperintense area consistent with periappendiceal edema. A small hypointense focus within the lumen suggests an appendicolith (dotted arrow). B) Coronal STIR sequence displaying the appendix (arrow) with similar finding. Image Credit: The figure is original and derived from the data collected in the present study.

Figure 3 shows one of the MRI scans from our study, highlighting its usefulness in assessing acute appendicitis. The use of sequences such as T2-weighted and STIR enhances the ability to detect signs of inflammation, such as oedema or swelling, and to spot associated findings such as appendicoliths. It allows for detailed soft tissue assessment while avoiding radiation exposure, making it especially valuable in select patient groups.

Ultrasound (US) findings

A total of 52 US scans were performed in the study, and the results showed the lowest sensitivity of all the three modalities. US had a sensitivity of 0.56 (95% CI, 0.41-0.70) and a PPV of 0.93. Around 45% of appendicitis cases were missed, later being picked up by CT, MRI, or found after surgery on a histopathology report. TN, FP and hence Sp and NPV, were not adequately powered for analysis due to a low number of patients in these groups (Table 3).

**Table 3:** Summary of ultrasound (US) scan results.

Ultrasound Findings	Number of Cases / Value
Total US scans	52
True Positives (TP)	27
False Negatives (FN)	21
↳ Confirmed later by CT	12
↳ Confirmed later by MRI	6
↳ Confirmed by Histopathology	3
True Negative (TN)	2
False Positive (FP)	2
Sensitivity (%)	56.25

A more thorough analysis of the patients who underwent an initial US scan was conducted to understand how US performs across different age groups because in real practice, it is not used the same way for everyone. In the 52 cases analysed, the female-to-male ratio was fairly balanced at 1:1.16. The average age in males was 21.42 years, while in females, it was 27.83 years. About one-third of the US group were under 18 (n=18), with the rest split between young adults (n=17) and older patients (n=17). US performed best in children under 18, with a sensitivity of 80%. In contrast, sensitivity appeared noticeably lower in adults, particularly in the 18-29 age group (Table 4).

**Table 4:** Summary of ultrasound (US) scans categorised by various age groups.

Age Group	Number of Patients	Sensitivity (%)	Sensitivity After Excluding Non-visualised Appendix (%)
< 18 years	18	80	85.71
18–29 years	17	37.5	75
≥ 30 years	17	52.94	100
Total	52	56.25	87.09

About a third of the cases (n=18) with negative US reports for appendicitis were later found to be due to the appendix not being visualised clearly, including one case (n=1) where excess tenderness limited assessment. Because of this, the scope of the study was expanded to assess the true sensitivity of US, equating only the cases where the appendix was clearly visualised. Out of the total 52 patients who initially had a US for suspected appendicitis, only 34 (65%) had a clearly identifiable appendix on their scans. Two cases under 18 years, eight cases aged 18-29 years, and eight cases aged 30 years or above did not have an identifiable appendix on their scans. When these cases with non-visualised appendices were excluded from analysis, US sensitivity improved from 0.56 to 0.87, a difference that was statistically significant (p=0.0059). The sensitivity also improved across different age groups after this adjustment (Table 4).

**Figure 4:**
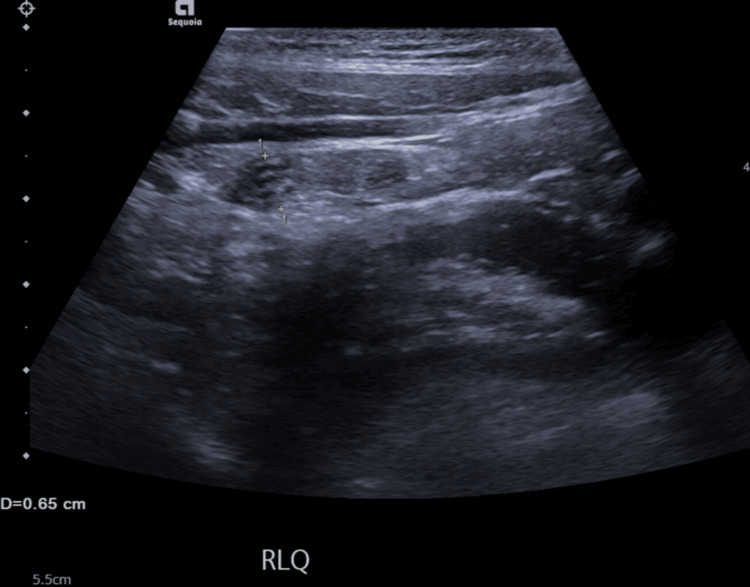
Ultrasound scan of a 12-year-old female from the study, where the right lower abdomen shows a blind-ending tubular structure measuring 6.5 mm, consistent with a dilated appendix. Typical ultrasonographic findings of appendicitis include a diameter ≥6 mm, wall thickening, absent peristalsis, and periappendiceal fat changes. Image Credit: The figure is original and derived from the data collected in the present study.

Failure to see the appendix on US was thought to be due to several factors, including overlying bowel gas, abdominal tenderness limiting probe pressure, high body mass index, or an unusual appendix position. These factors can reduce the diagnostic value of US despite its advantages. A US scan from within the study, which shows a dilated appendix visualised in the right lower quadrant, is shown in Figure 4. The grainy, low-resolution, two-dimensional grayscale image highlights the limitations of US, namely, its restricted soft tissue contrast, which can affect diagnostic confidence.

## Discussion

Several imaging modalities are used for the diagnosis of appendicitis. Each has its strengths and limitations, and the ideal choice often depends on availability, patient factors, and clinical urgency. This section discusses the diagnostic performance, practical challenges, and broader implications of CT, MRI, and US scans in this setting.

CT scans

CT continues to outperform in both sensitivity and negative appendectomy rates. In our sample, CT had a sensitivity of approximately 99% and a negative appendectomy rate of 2.24%, which are at par with the standards set out by the Royal College of Radiologists, UK (target sensitivity of >90% and negative appendectomy rates <10% for CT scans) [2]. An 18-year-long prospective study conducted by Raja et al. and published in 2010 noticed an upward trend in utilising a pre-operative CT scan in patients undergoing surgeries for appendicitis over the years [3]. This trend of rising usage of CT scans was also associated with falling negative appendectomy rates from 23.0% to 1.7%, reducing the number of unnecessary surgeries significantly from the annual number of 217 per year to 119 per year [3]. These findings reinforce the value of CT as a reliable tool in optimising surgical decision-making and reducing avoidable operations. Despite its strengths, CT is not without its challenges. Some of them are logistical, but others are related to safety.

High Demand and Long Waiting Times

One of the most pressing challenges in many hospitals is simply the high demand and limited availability of CT scanners, which often results in long wait times. A large study conducted in a big public emergency department by Lee et al. highlights some of the setbacks with CT being so popular for emergencies. On studying 38,359 scans retrospectively in a single large hospital from March 2023 to February 2024, they found that the mean time to complete a CT scan was two hours 34 minutes for contrast scans and one hour 46 minutes for non-contrast scans [4]. These hours quickly add up if the hospital is a big tertiary hospital receiving patients of major surgical or trauma cases, including other emergencies from throughout a big metropolitan city, leading to big waiting queues for CT scanners. The waiting times significantly increase if you account for the time taken in preparation and positioning, transport logistics and coordination between departments, etc. The process of obtaining a CT within the ED, from ordering to scan completion, is highly complex, with multiple potential sources of delay.

Shortage of Scanners and Specialists

This demand is compounded by a nationwide shortage of radiologists and limited scanner infrastructure, especially in the UK. There is a documented nationwide shortfall of radiologists in the UK. According to a report in the 2023 clinical radiology and clinical oncology workforce census by The Royal College of Radiologists, the UK had a 30% shortfall in clinical radiology consultants, equating to 1,962 fewer doctors than needed [5]. This gap is projected to rise to 40% (3,670 doctors) by 2028 [5]. In 2014, the UK had one of the lowest numbers of CT scanners, ranking second lowest among 31 countries, which was 8.03 scanners per million population, one study observed [6]. The National Health Service (NHS) employs traditional approaches, such as outsourcing, insourcing, and hiring locum radiologists, to address the workforce gap. These methods have often led to increased financial burdens without providing sustainable, long-term solutions [6].

Radiation Exposure and Cancer Burden

CT scans use ionising radiation, which increases cancer risk. While the individual risk is low and often outweighed by diagnostic benefits, the cumulative effect across the population is significant. A modelling study published in JAMA Internal Medicine estimated that the 93 million CT scans performed in the United States in 2023 could result in over 100,000 future cancer cases, including around 9,700 in children [7,8]. Most of these were linked to abdominal and pelvic CTs in adults (37,500 cases) and CT head scans in children (5,100 cases), with common cancers including lung (22,400), colon (8,700), leukaemia (7,900), and bladder (7,100) [8]. Children and adolescents face higher estimated risks [7]. In contrast, the UK performs fewer than 100 CT scans per 1,000 people annually, well below the US rate of over 250 per 1,000, largely due to stricter regulations. These ensure that scans are clinically justified and doses are tailored based on age and organ sensitivity, with radiologists often reviewing each case beforehand [7]. Further supporting the concern, a large Australian study in 2013 involving over 680,000 young people found a 24% higher cancer risk in those who had CTs, with risk increasing by 16% per additional scan. Younger age groups had stronger associations [9]. Similarly, a 2021 review in the British Journal of Radiology covering 17 studies found that increased radiation exposure from CT was consistently linked to higher rates of cancers such as leukaemia and brain tumours [10]. While the absolute risk remains small, these findings highlight the need for careful and considered CT use - especially in children [10].

CT remains a highly accurate tool in diagnosing appendicitis, helping reduce unnecessary surgeries. However, its growing demand, limited availability, and radiation risks, especially in younger patients, highlight the need for balanced, thoughtful use. The challenge now is not just using CT well, but using it wisely.

MRI scans 

MRI remains an extremely reliable diagnostic tool in patients with suspected acute appendicitis, including outstanding results in adults, children, and pregnant women. MRI typically uses sequences, such as T2-weighted and diffusion-weighted imaging to visualise the appendix and surrounding inflammation. Findings suggestive of appendicitis include appendiceal dilation, wall thickening, peri-appendiceal fat stranding, and fluid collections [11].

MRI is especially valuable when evaluating suspected appendicitis in patients where radiation should be avoided, mainly children and pregnant women. Published in 2021, a large meta-analysis of various studies in large university hospitals of Europe and North America discovered comparable results. On analysing 58 studies and a total of 7462 participants, MRI showed excellent results with overall sensitivity (Sn) and specificity (Sp) of 0.95 and 0.96, respectively. These results were consistent in all three populations analysed (i.e., adults, paediatric population, and pregnant women) [12]. The accuracy of MRI remained high even after studies of high or unclear risk of bias were excluded. This trend is also reflected in our observation, where MRI demonstrated a very high sensitivity (Sn=1.0), although the sample size was small (n=6), likely due to its lower preference and limited availability in emergency settings. While the accuracy looks great on analysis, the numbers are just too small to rely on, so this group is underpowered for any proper analysis.

MRI has a high sensitivity and specificity for diagnosing acute appendicitis, comparable to CT, but without the associated radiation risk. Although it serves as an excellent alternative when CT is contraindicated, particularly in pregnancy and paediatric cases, MRI is not typically used as a first-line investigation due to several practical limitations. Access to MRI can be limited, especially in emergency settings where rapid decision-making is required. It is not always readily available out of hours, and the scan itself takes longer to perform and interpret compared to other modalities. These delays can affect timely management, especially in acute settings where speed is critical. Additionally, MRI is more expensive and may not be feasible in all healthcare environments, which further limits its routine use.

US scans

Our findings align with previous multicentre studies evaluating US in diagnosing appendicitis, with it showing the lowest sensitivities of the three modalities (Sn=0.56). A UK-based retrospective study involving 573 patients showed that the appendix was not visualised in nearly half of the scans, resulting in a sensitivity of 81.7% and specificity of 53.9%. When non-visualised appendices were grouped with negative scans, sensitivity dropped significantly to 51.8%, though specificity improved to 81.4% [13]. Similarly, a paediatric-focused multicentre study reported an overall US sensitivity of 72.5% and specificity of 97.0%, with sensitivity rising to 97.9% when the appendix was seen [14]. Sensitivity appeared to improve with more frequent use (i.e., centres where more US scans were performed had better outcomes) [14]. In our study, the overall sensitivity of US increased to 87% where the appendix was identified. Improvement across different age groups was noticed, sensitivity rising to 85% in < 18 years, 75% in 18-29 years, and 100% in > 30 years.

These findings highlighted that the diagnostic performance of US is strongly influenced by the ability to visualise the appendix, which varies widely between centres and operators. Visualisation rates of the appendix with US vary widely in the published literature, from as high as 98% to as low as 22% [14]. A study highlighted that, while US is a valuable diagnostic tool, its effectiveness can be influenced by factors such as patient body habitus and the ability to visualise the appendix. These limitations can lead to variability in diagnostic performance [15]. In our study, where around 45% of appendicitis cases were missed by the US, most of them (85%) were subsequently picked up by further imaging. Further imaging, when performed in patients with an unidentifiable appendix on ultrasound, children under 18 were not offered any CT scans; MRI was preferred for this group. In the older age groups, follow-up imaging included a mix of CT and MRI, with a general preference for CT over MRI.

The main benefit of US is its non-invasive nature, accessibility, and absence of radiation, making it a practical first-line investigation in appendicitis evaluation. However, the variable reported sensitivities and frequent inability to visualise the appendix remain key drawbacks, leading to potential diagnostic uncertainty. Other diagnostic modalities should be considered when the appendix is not definitively visualised by US.

Diagnostic approach and the role of imaging in patients with suspected appendicitis

**Figure 5:**
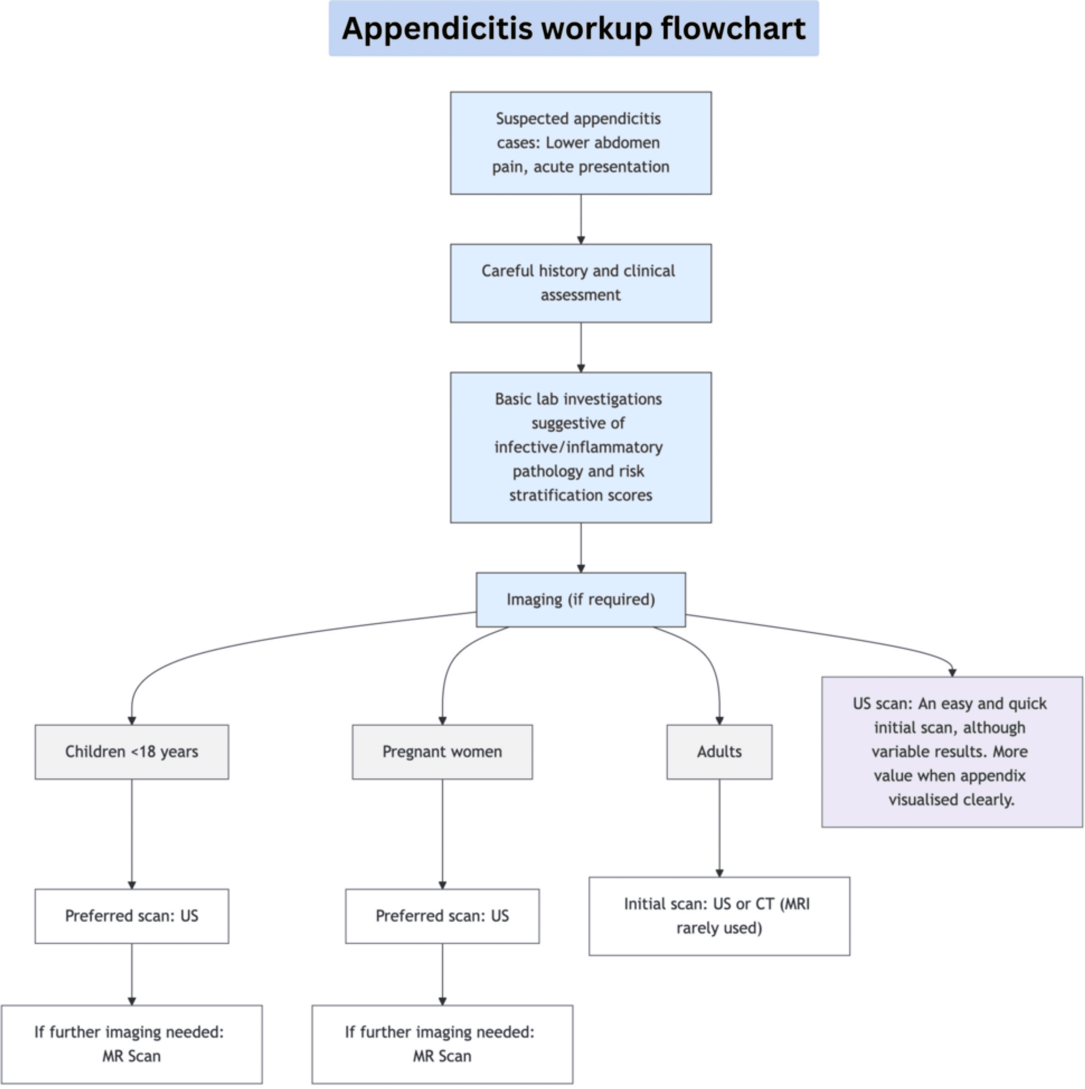
Appendicitis workup flowchart representing the flow of the appendicitis work-up observed in this study. CT: computed tomography; MR: magnetic resonance scan; US: ultrasound scan Image Credit: The figure is original and derived from the data collected in the present study.

The starting point for diagnosing appendicitis is still clinical suspicion. It should be high in patients presenting with typical signs (i.e., abdominal pain starting centrally and moving to the right iliac fossa, along with nausea, anorexia, or vomiting). Signs of sepsis or shock should raise concerns for perforation, which requires urgent attention. In pregnant women, appendicitis is the most common non-obstetric surgical emergency, and delays can seriously impact both mother and fetus [16]. The figure below summarises the diagnostic pathway observed in the cases studied, beginning with history-taking, clinical assessment, followed by supportive laboratory findings and progressing to imaging, with modality selection based on age, patient status, and clinical scenario (Figure 5).

A separate Dutch nationwide audit involving 1,975 adult patients across 62 hospitals found that nearly all received preoperative imaging, leading to a low negative appendectomy rate of 2.2%. This underlines the benefit of routine imaging in reducing unnecessary surgeries [17]. Clinical scoring systems such as the Alvarado, Appendicitis Inflammatory Response (AIR), and Adult Appendicitis Score (AAS) help stratify risk, especially in uncertain cases, but they are not definitive on their own [18]. Their sensitivity and specificity vary, particularly in primary care, so they should be used alongside clinical judgement and imaging [19,20]. In children, a large review showed that no single history, physical examination, laboratory finding, or score attained on the Paediatric Appendicitis Score (PAS) can eliminate the need for imaging studies. A positive point-of-care US (POCUS) increases the likelihood of appendicitis, but a negative result does not rule it out. It is helpful for confirmation but should not replace a full clinical and imaging assessment [21].

In conclusion, diagnosing appendicitis continues to depend heavily on clinical assessment, with support from scoring tools, imaging, and basic investigations. Imaging plays a valuable role, but its use should be based on the individual clinical scenario. Before deciding on a scan, it is important to consider the strengths of each modality and how accurate and useful the results are likely to be. Practical challenges such as delays in arranging imaging, time constraints, radiation exposure, and access to advanced modalities also need to be factored in. Ultimately, imaging should enhance clinical judgement, not replace it, and should be used thoughtfully alongside other key diagnostic steps.

Limitations

This study has several limitations. First, its retrospective design inherently introduces selection and information biases. Additionally, we did not include patients who underwent appendectomy without prior imaging. While many of these patients likely had clear clinical signs, their exclusion prevents assessment of whether imaging could have contributed to reducing unnecessary surgeries. Inter-operator variability in US interpretation was also not accounted for, referring to differences in technique, skill level, and experience among operators. This variation may have contributed to the lower sensitivity observed in certain subgroups and can be considered a form of observer-dependent diagnostic variability, a known source of error in imaging-based studies. Furthermore, the number of MRI scans included was too small to allow for meaningful analysis or confident conclusions. The limited use of MRI in this setting also highlights the logistical and resource-related challenges. Specificity, PPV, and NPV were not reported for every imaging modality and, if reported, were only included in sections where sample sizes were sufficient to allow meaningful interpretation. Finally, although we compared US sensitivity across different age groups, the statistical test results comparing the age groups were not included due to small sample sizes, which limits the reliability and statistical power of that comparison. These limitation reflects the study’s real-world approach, which aimed to capture actual imaging practices rather than assess each modality in depth.

## Conclusions

The study included 130 cases, with 91 CT, six MRI, and 52 US scans. CT and MRI proved to be the most sensitive modalities; however, the use of MRI was limited due to practical limitations. US was the second most used modality after CT, and it had an overall sensitivity of 0.56 in the cases observed. It should be the first-line scan in children, pregnant women, and in situations where radiation exposure is a concern. Although its sensitivity varied across the groups analysed, it performed best in patients under 18 years. It is widely accessible, safe, and quick, but its reliability is limited when the appendix is not visualised clearly. The appendix was not seen in nearly one-third of the cases in this study; when these non-visualised cases were excluded, US sensitivity improved significantly. It rose from 0.56 to 0.87, indicating that the results are more reliable when the appendix is clearly visualised. CT scans showed excellent diagnostic performance, with a sensitivity of 0.98 and a low negative appendectomy rate. They are faster than MRI and readily available in most centres, but concerns remain about radiation exposure, particularly in younger patients, and especially when considering cumulative exposure over time. Additionally, scanner availability and demand pressures in busy hospitals often lead to delays, something to consider when ordering the scans. MRI achieved perfect sensitivity (1.0) in our small sample and offers a radiation-free alternative. However, due to cost, scan time, and limited emergency access, MRI remains a broadly underutilised modality overall.

In practice, imaging decisions should be tailored to the clinical scenario and patient group. We start with US when feasible, especially in children and pregnant women, and escalate to CT when US is inconclusive or if urgency requires. MRI should be considered selectively, particularly in situations where radiation exposure must be avoided, such as in children and pregnant women, and when time permits. Imaging should support, not replace, sound clinical judgement, with the overall aim of improving diagnostic certainty while minimising unnecessary intervention and harm.
